# Radioprotective Effect of Lidocaine on Neurotransmitter Agonist-Induced Secretion in Irradiated Salivary Glands

**DOI:** 10.1371/journal.pone.0060256

**Published:** 2013-03-29

**Authors:** Yu-xiong Su, Geza A. Benedek, Peter Sieg, Gui-qing Liao, Andreas Dendorfer, Birgit Meller, Dirk Rades, Matthias Klinger, Samer G. Hakim

**Affiliations:** 1 Department of Oral and Maxillofacial Surgery, University of Luebeck, Luebeck, Germany; 2 Department of Oral and Maxillofacial Surgery, Guanghua School of Stomatology, Sun Yat-sen University, Guangzhou, China; 3 Walter-Brendel-Centre of Experimental Medicine, Ludwig-Maximilians-University Munich, Munich, Germany; 4 Department of Radiology and Nuclear Medicine, University of Luebeck, Luebeck, Germany; 5 Department of Radiation Oncology, University of Luebeck, Luebeck, Germany; 6 Institute of Anatomy, University of Luebeck, Luebeck, Germany; National Institute of Dental and Craniofacial Research, United States of America

## Abstract

**Background:**

Previously we verified the radioprotective effect of lidocaine on the function and ultrastructure of salivary glands in rabbits. However, the underlying mechanism of lidocaine's radioprotective effect is unknown. We hypothesized that lidocaine, as a membrane stabilization agent, has a protective effect on intracellular neuroreceptor-mediated signaling and hence can help preserve the secretory function of salivary glands during radiotherapy.

**Methods and Materials:**

Rabbits were irradiated with or without pretreatment with lidocaine before receiving fractionated radiation to a total dose of 35 Gy. Sialoscintigraphy and saliva total protein assay were performed before radiation and 1 week after the last radiation fraction. Isolated salivary gland acini were stimulated with either carbachol or adrenaline. Ca^2+^ influx in response to the stimulation with these agonists was measured using laser scanning confocal microscopy.

**Results:**

The uptake of activity and the excretion fraction of the parotid glands were significantly reduced after radiation, but lidocaine had a protective effect. Saliva total protein concentration was not altered after radiation. For isolated acini, Ca^2+^ influx in response to stimulation with carbachol, but not adrenaline, was impaired after irradiation; lidocaine pretreatment attenuated this effect.

**Conclusions:**

Lidocaine has a radioprotective effect on the capacity of muscarinic agonist-induced water secretion in irradiated salivary glands.

## Introduction

Salivary gland dysfunction is one of the major side effects of radiotherapy in patients treated for head and neck cancer [1]. The exact mechanism of radiation-induced salivary gland injury *per se* remains elusive. Although radiation induced sterilization of progenitor cells which prevents the replenishment of saliva-producing cells is considered as the main cause for late-stage effects [2], [3], the mechanism of early-stage functional damage is different [2], [4]. Salivary acinar cells are well differentiated and have a low mitotic index [2]. Previous studies found that after radiation the saliva flow reduced rapidly [5], [6]. Secretory activity of salivary glands decreased to 50% of normal levels whereas less than 3% of apoptosis activity was observed 3 days after radiation [5] and that no significant cell loss was observed within 10 days after radiation [7]. This rapid radiation induced changes is not compatible with mitotic or apoptotic cell death and that an alternative hypothesis of early-stage radiation damage to the salivary glands is needed [4], [7], [8].

Recently, researchers demonstrated that receptor-effector signal transduction of water secretion in salivary glands was impaired after irradiation [4], [7], [9]. Coppes et al. [4] suggested that radiation impairs the muscarinic acetylcholine agonist-induced activation of protein kinase C-alpha, measured as its translocation to the plasma membrane, leading to damage in the ability to mobilize calcium from intracellular stores, which is the driving force for water secretion. Accordingly, it was proposed that the plasma membrane is the key site of early-stage radiation damage to the salivary glands and that the damage of intracellular neuroreceptor-mediated signaling contributes to secretion dysfunction [8]. This new concept of the mechanism of radiation-induced salivary gland injury leads to promising, novel possibilities for interference with and prevention of this early-stage damage.

Prompted by a finding stabilizing the basolateral membrane using prophylactic lidocaine reduced radiation-induced damage in cultured parotid acinar cells [10], we recently verified that lidocaine was able to prevent radiation-induced damage to the function and ultrastructure of salivary glands *in vivo* in rabbits [1], [11]. However, the pharmacological mechanism of lidocaine's radioprotective profile is still largely unknown. Therefore, we hypothesized that lidocaine, as a membrane stabilization agent, has a protective effect on intracellular neuroreceptor-mediated signaling and hence can help preserve the secretory function of salivary glands during radiotherapy. To test this hypothesis, we investigated the salivary glands of control, irradiated/sham-treated, and irradiated/lidocaine-pretreated rabbits by stimulation with neurotransmitter agonists in isolated glandular acini and evaluated the simultaneous functional impairment of related salivary glands using sialoscintigraphy and saliva protein assay.

## Materials and Methods

### Animals and study design

The study was approved by the Department of Landscape, Environment and Rural Areas, Kiel, Germany (permit number: V 312-72241.122-14) according to the current German law on the protection of animals. Twenty-four adult Chinchilla Bastard rabbits were randomized into unirradiated control, irradiated/sham-treated, and irradiated/lidocaine-pretreated groups of 8 rabbits each. All *in vivo* procedures were performed with the rabbits under general anesthesia induced using a combination of 3 mg/kg (S)-ketamine hydrochloride and 0.1 mg/kg xylazine hydrochloride. In the irradiated/sham-treated and irradiated/lidocaine-pretreated groups, rabbits underwent a first sialoscintigraphy and then received one fraction of radiation each day for 7 days. For each rabbit in the irradiated/lidocaine-pretreated group, lidocaine hydrochloride (Xylocaine, 2%, 10 mg/kg) was slowly administered into a marginal ear vein 10 minutes before each radiation fraction. One week after the last radiation fraction, a second sialoscintigraphy was performed. Whole stimulated saliva was collected during sialoscintigraphy for the saliva total protein assay. A biopsy specimen (5×5×5 mm^3^) was excised from the parotid tissue immediately after the second sialoscintigraphy, and the acini were isolated for neurotransmitter agonist stimulation.

### Radiation

X-ray irradiation was performed as described previously [1], [11], using MEVATRON 74-Siemens teletherapy unit (photon energy) operated at 10 MeV with a dose rate of 3 Gy/min. The output of accelerator was 1 Gy = 80 MU at source-to-skin distance of 98 cm. A single dose of 5 Gy was applied during each irradiation. An axial beam was directed toward the head of the rabbit, extending from the retroauricular region into the tip of the nose (field size 7.5 ×10 cm), thus including all potential localization of salivary glands. [1], [11]Fractions were administered for 7 days, yielding a total dose of 35 Gy. The equivalent dose in 2-Gy fractions (α/β = 3 Gy for late damage) was 56 Gy. [12]Since an equivalent dose in 2-Gy fractions of 50 Gy is associated with a 100% risk of relevant xerostomia (Tolerance Doses 100/5)[13], our study design reflected a clinically relevant situation.

### Sialoscintigraphy

Rabbits were injected intravenously with 100 MBq of pertechnetate (99mTcO_4_) and then imaging was done immediately with a triple-head gamma camera (Picker 3000XP, LEUHR collimator; Philips). Twenty minutes after injection, saliva production was stimulated by subcutaneous administration of 0.01 mg/kg carbachol (Sigma-Aldrich, Seelze, Germany), and then the secretory phase was monitored for 25 minutes. The percentage of uptake of the administered activity was calculated for the 10 to 19 minutes and 36 to 45 minutes. The salivary ejection fraction was defined as a percentage: maximum uptake (until the 20th minute)−remaining activity (after 45 minutes)/maximum uptake of the gland.

### Saliva total protein assay

Saliva collections were performed during scintigraphy. After carbachol stimulation at the 20th minute of scintigraphy, saliva was collected for 25 minutes, until the end of scintigraphy.

Immediately after collection, the saliva samples were stored at –70°C. Saliva total protein concentration ( µg/L) was measured using the enzyme-linked immunosorbent assay, performed as recommended by the manufacturer (R&D Systems, Minneapolis, MN).

### Isolation of glandular acini

In contrast to fully isolated parotid acinar cells, individual intact acini as secretory units were obtained to mimic *in vivo* secretion activity. The procedure for isolating glandular acini was based on modified lacrimal acini isolation protocols [14], [15]. Briefly, 5×5×5 mm^3^ of tissue were excised from the parotid gland and immediately immersed in a culture medium that contained Dulbecco's modified Eagle medium/Ham's F-12, L-glutamine, 5% fetal calf serum, and penicillin/streptomycin. After being cut into pieces, the samples were immersed in a culture medium that contained purified collagenase (Sigma-Aldrich, Seelze, Germany), 60 U/mL, and then incubated at 37°C with oxygen for 40 minutes with constant agitation (100 cycles/min). After incubation, moderate pipetting yielded isolated acini, which were then washed twice by centrifugation (3000 rpm, 5 minutes) and resuspended. An MTT assay was performed to evaluate the vitality of the isolated acini.

### Neurotransmitter agonist stimulation

Hundreds of acini could be isolated each time for every single gland. Isolated acini were loaded with Fluo-4 AM (Invitrogen, Paisley, UK) with a concentration of 4 µM in the culture medium by incubation for 60 minutes at 37°C with oxygen. The acini and culture medium were placed on the nonfluorescent coverslips coated with Cell-Tak (BD Biosciences, San Jose, CA) [14]. For every gland, the isolated acini were randomly placed on six coverslips and stimulated with neurotransmitter agonists. The acini on three of these coverslips were stimulated with 10 µM carbachol, and the acini on the other three were stimulated with 10 µM adrenaline (Sigma).

A laser scanning confocal microscope (TCS SP5; Leica Microsystems CMS GmbH, Mannheim, Germany) was used to conduct the kinetic study of Ca^2+^ influx in isolated acini. The main microscopy settings were pinhole: 600 µM; argon laser: 20% of maximum; water immersion objective: ×20; mode: xyt; and time interval: 10 seconds. For each acinus, Ca^2+^ influx in response to stimulation with agonists was measured with a single wavelength excitation technique. The fluorescence of Fluo-4 AM was measured with filters for excitation at 488 nm and for emission at 526 nm.

During confocal microscopy, we selected the field of view which included at least 3 acini on each slide so that they could be analyzed at the same time. The amounts of fluorescence before and after agonist stimulation of each selected acinus were recorded and quantitatively analyzed by LAS AF Lite 1.8 software (Leica Microsystems CMS GmbH).

### Ultrastructural study

Small pieces of biopsy samples were fixed with 5% glutaraldehyde in 0.1 M cacodylate buffer for 1 hour, postfixed in 1% OsO_4_ for 2 hours at 4°C, dehydrated, and embedded in epoxy resin. The ultrathin sections were stained with uranyl acetate and lead citrate and examined by transmission electron microscopy to evaluate basolateral membrane alterations.

### Statistics

All statistical analyses were performed using the SPSS software program (version 16.0; IBM Corporation, Armonk, NY). When proved to follow a normal distribution, values were expressed as mean ±SD. The means were then compared using the two-sided *t*-test for paired samples, and Least Significant Differences Test (LSD test) was used for multiple comparisons. An α level of *p*<0.05 was considered statistically significant.

## Results

The animals tolerated radiation well and continued to eat and drink as usual. Mild mucositis was observed as early as when fractions were administered for 5 days and lasted for about two weeks. No major radiation complications were found. No significant loss of body weight was observed.

### Effect of radiation on saliva secretion function

**Figure 1:**
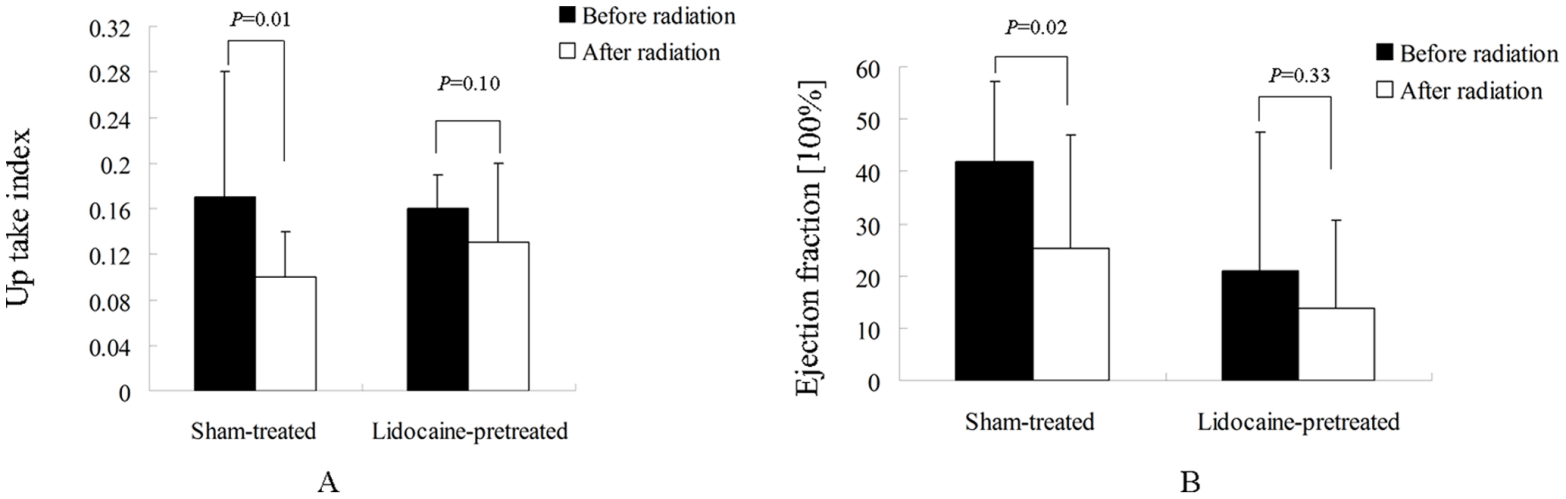
Scintigraphic results of parotid glands before and one week after irradiation. (A) Uptake index. (B) Ejection fraction.

One week after irradiation, the parotid glands in the irradiated/sham-treated rabbits displayed a significant reduction of primary uptake and of excretion fraction compared with values measured before irradiation (*p* = 0.01 and *p* = 0.02, respectively). In contrast, the glands in the irradiated/lidocaine-pretreated group displayed no significant difference in primary uptake or excretion fraction after irradiation (*p* = 0.10 and *p* = 0.33, respectively).(Figure 1) This result indicates that radiation impairs both the primary tracer uptake and the ejection function of the parotid glands but that pretreatment with lidocaine protects against these effects and thus can preserve the glands' saliva secretion function.

### Effect of radiation on saliva total protein

**Figure 2:**
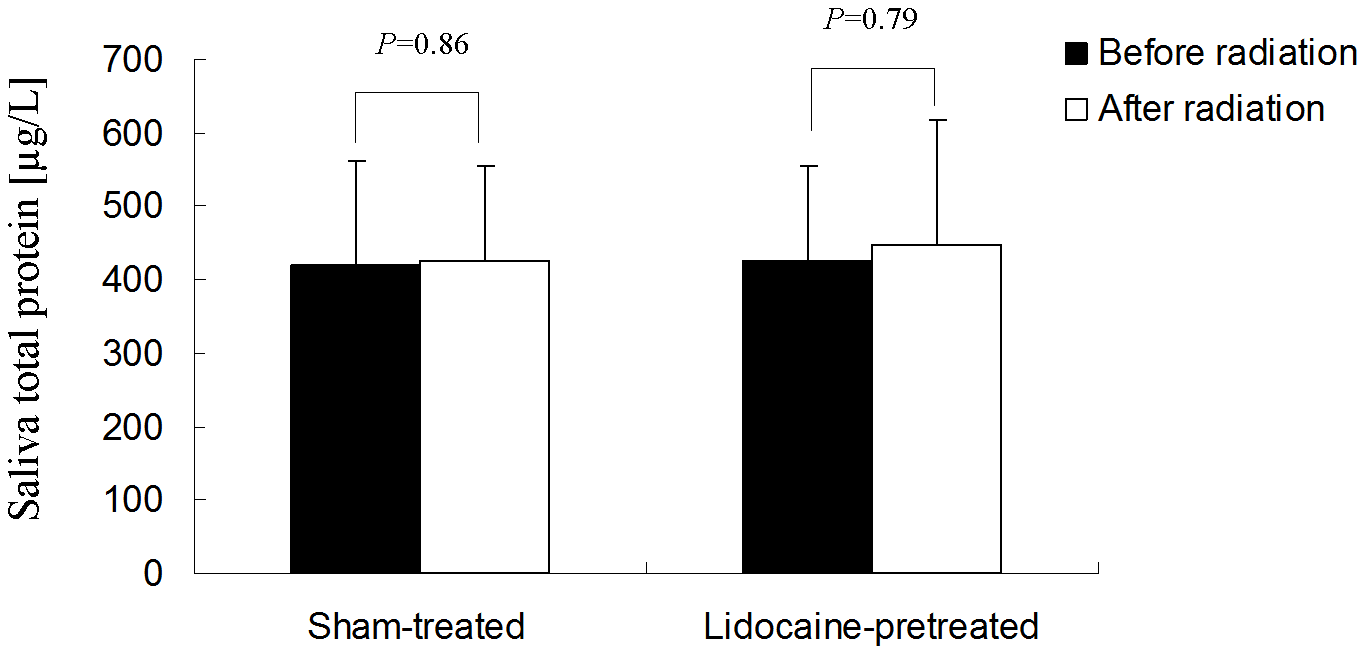
Saliva total protein concentration before and one week after irradiation.

For both the irradiated/sham-treated and the irradiated/lidocaine-pretreated rabbits, the two-sided paired-sample *t*-test showed no significant difference between protein concentrations before and after irradiation (*p* = 0.86 and *p* = 0.79, respectively) (Figure 2). Consequently, saliva total protein secretion was not affected 1 week after irradiation, and lidocaine did not affect this parameter in either irradiated group.

### Effects of neurotransmitter agonists on the Ca^2+^ dynamics of acinar secretion

**Figure 3:**
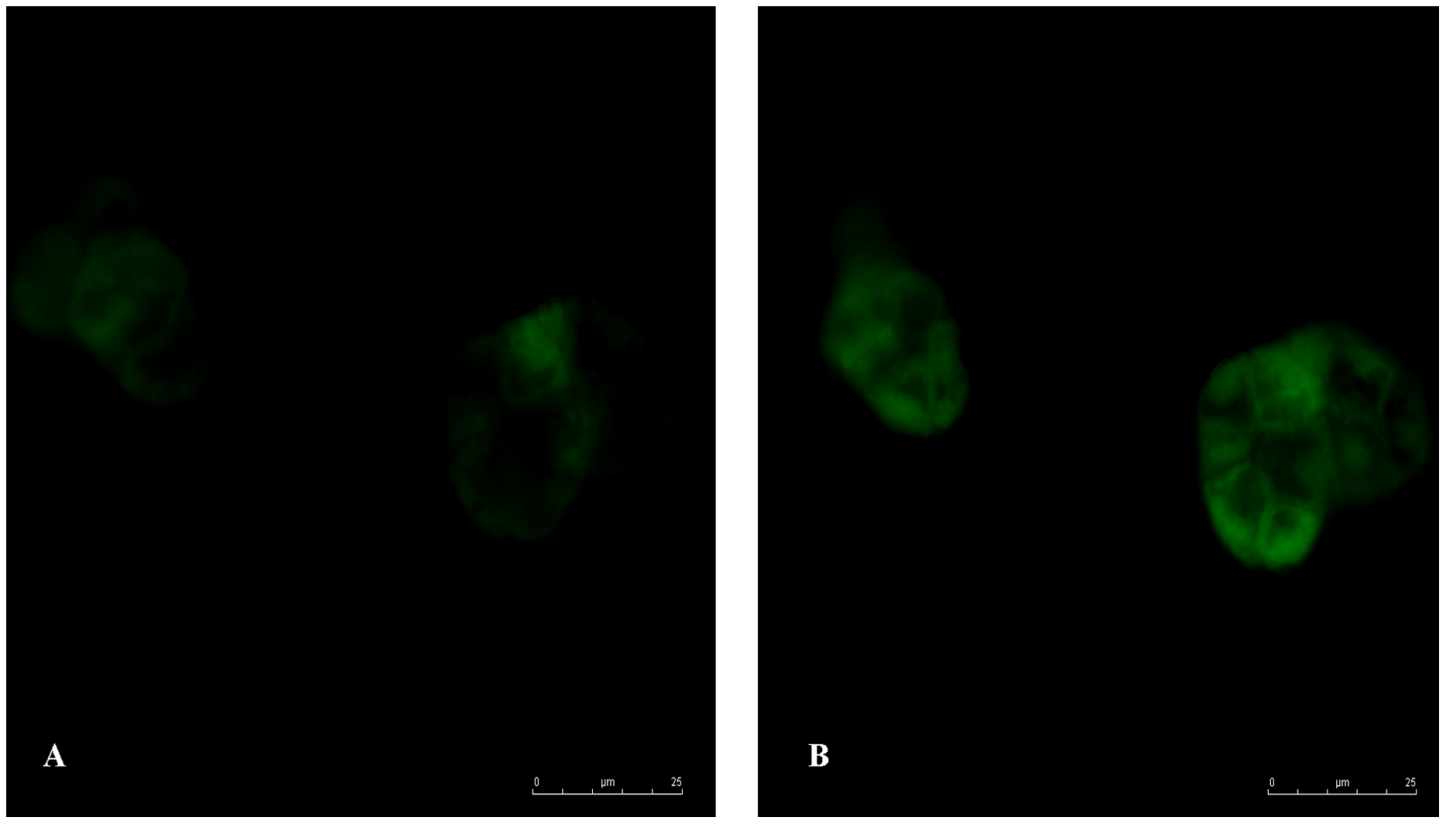
Confocal microscopic view of isolated acini loaded with Fluo-4 AM. (A) Before carbachol stimulation; (B) After carbachol stimulation.

**Figure 4:**
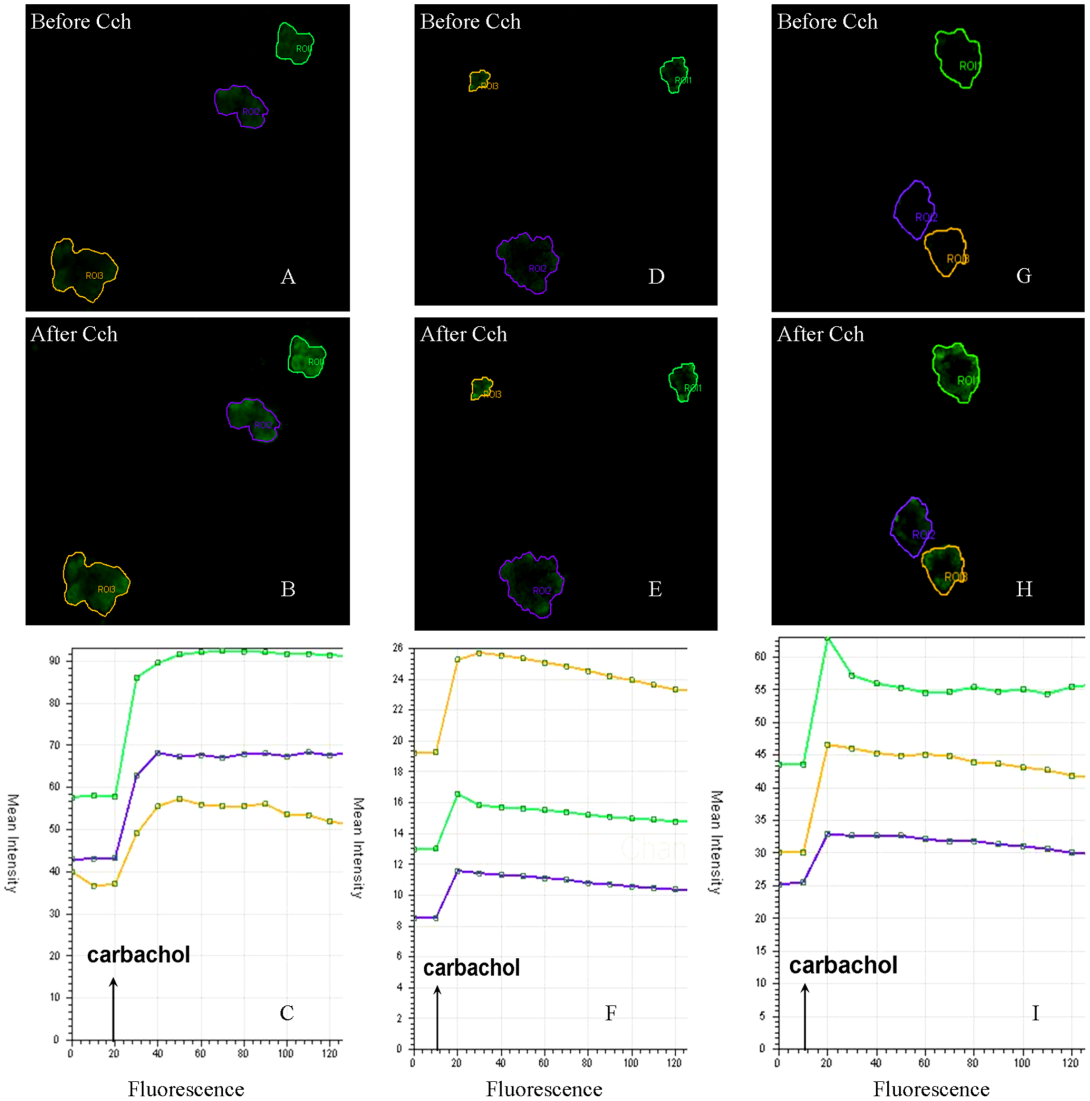
Confocal microscopic study of fluorescence alteration before and after carbachol stimulation of the acini. (A-C) Rabbit No. 3 in the control group; (D-F) Rabbit No. 3 in the irradiated/sham-treated group. (G-I) Rabbit No. 3 in the irradiated/lidocaine-pretreated group. (A, D and G) Laser scanning confocal microscopic views of regions of interest before stimulation. (B, E and H) Laser scanning confocal microscopic views of regions of interest after stimulation. (C, F and I) Quantitative dynamic analysis of fluorescence. Cch: carbachol.

**Figure 5:**
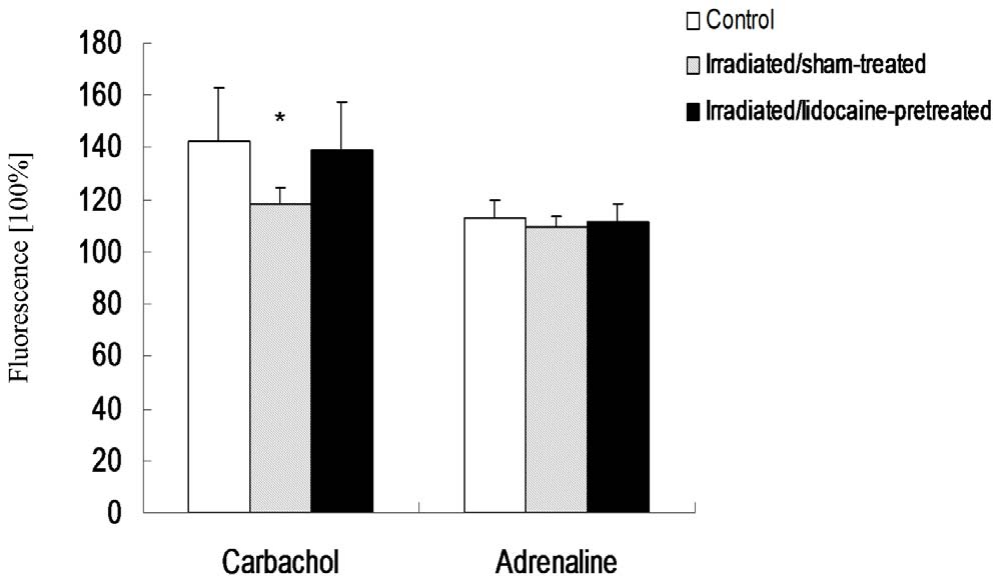
Fluorescence after neurotransmitter agonist stimulation. The level of fluorescence prior to agonist stimulation was set as 100%. * indicates significant change when compared to other two groups.

After isolation the acini appeared intact (Figure 3). The MTT assay verified the vitality of isolated acini. Alteration of fluorescence after neurotransmitter agonist stimulation of the selected acini was quantitatively analyzed (Figure 4). The level of fluorescence prior to agonist stimulation was set as 100%. After carbachol stimulation, the mean±SD fluorescence levels in the control, irradiated/sham-treated, and irradiated/lidocaine-pretreated groups were 142.2±20.9%, 118.1±6.6%, and 138.9±18.6%, respectively. LSD test showed that the irradiated/sham-treated group had significantly less fluorescence alteration than the control group and the irradiated/lidocaine-pretreated group (*p* = 0.01 and *p* = 0.02, respectively). The fluorescence alterations of the control and irradiated/lidocaine-pretreated groups did not significantly differ (*p* = 0.69). (Figure 5) Together, these results suggest that Ca^2+^ influx is attenuated in response to carbachol in irradiated parotid acini, which indicates impairment of intracellular muscarinic receptor-mediated signaling of the acini; pretreatment with lidocaine, however, has a radioprotective effect on the acini's capacity of muscarinic agonist-induced secretion.

After adrenaline stimulation, the mean±SD fluorescence levels in the control, irradiated/sham-treated, and irradiated/lidocaine-pretreated groups were 113.2±6.6%, 109.6±4.0%, and 111.9±6.8%, respectively. LSD test indicated similar fluorescence alterations in the irradiated/sham-treated and irradiated/lidocaine-pretreated groups when they were compared with the control group (*p* = 0.237 and *p* = 0.657, respectively) (Figure 5). These results reveal that adrenergic agonist-induced secretion was not impaired one week after irradiation.

### Effects on tissue ultrastructure

**Figure 6:**
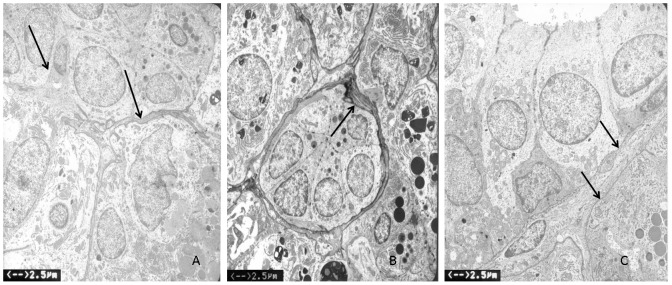
Effects on tissue ultrastructure examined by transmission electron microscopy. (A) Normal basolateral membrane in the control group (arrow). (B) In the irradiated/sham-treated group, thickening and irregular plump formations of basolateral membrane were observed (arrow). (C) In the irradiated/lidocaine-pretreated group, no obvious basolateral membrane alteration was observed (arrow).

In all irradiated/sham-treated rabbits, alterations of the basolateral membrane (thickening and irregular plump formations) were observed. In contrast, no obvious basolateral membrane alteration was observed in the irradiated/lidocaine-pretreated group (Figure 6).

## Discussion

On the basis of our results, we conclude that lidocaine, as a membrane stabilization agent, has a protective effect on the capacity of muscarinic agonist-induced water secretion of acini and hence can help preserve the secretory function of salivary glands during radiotherapy. To our knowledge, this is the first investigation of the radioprotective effect of lidocaine on neurotransmitter agonist-induced secretion in irradiated salivary glands.

Many prophylactic modalities have been suggested to increase the tolerance of salivary gland tissue to radiation; such methods include depletion of secretory granula by muscarinic alkaloid pilocarpine [16], pretreatment with the free-radical scavenger amifostine [17], increasing proliferation of acinar and intercalated ductal cells by pilocarpine [2], and stem cell therapy to replace damaged cells [18]; in addition, aquaporin-1 gene transfer which led to increased fluid secretion from surviving duct cells has been tested [19], [20]. In our previous studies we found a prevailed radioprotective effect of lidocaine compared with amifostine and pilocarpine based on functional, immunohistochemical, and ultrastructural investigations [1], [11]. In the present study, we further elucidated the potential pharmacological mechanism of the radioprotective effect of the membrane-stabilizing agent lidocaine.

The muscarinic-cholinergic receptors in acinar cells mediate the primary fluid secretory response [21]. When the agonists bind to the muscarinic receptors, a Gq-protein-mediated activation of plasma membrane-bound phospholipase C is elicited. As a result, membrane-bound phosphatidylinositol 4,5-bisphosphate (PIP_2_) is hydrolyzed into diacylglycerol and inositol 1,4,5-trisphosphate (IP_3_). Afterwards, IP_3_ binds to an intracellular receptor, mobilizing Ca^2+^ from internal Ca^2+^ stores such as endoplasmic reticulum [4], [21]. Thus, an increase in Ca^2+^ mobilization is the predominant mechanism of primary saliva secretion in acini [4], [8]. In the present study we found that stimulation with carbachol induced an increase in Ca^2+^ influx in the acini, but this carbachol-induced influx was diminished in glands functionally impaired by irradiation. This result is consistent with a previous investigation of isolated individual acinar cells [4]. The most novel finding of our study is the evidence that the radioprotective feature of lidocaine may be due to its ability to protect carbachol-induced Ca^2+^ influx in acini.

It is known that an increase in the Ca^2+^ concentration induces the opening of a Ca^2+^ -activated anion (Cl^–^ and HCO_3_^–^) channel and the water channel aquaporin-5 in the apical membrane as well as a K^+^ channel and a Na^+^/K^+^/2Cl^–^ cotransporter in the basolateral membrane [21]. Ultimately, the binding of agonists to receptors leads to secretion of isotonic primary saliva. 99mTcO_4_ used as a radiotracer in our study shares the Na^+^/K^+^/2Cl^–^ cotransporter localized to the basolateral acinar cell membrane [22], which consequently affects the initial tracer uptake of salivary glands. This relationship explains both our scintigraphic results after irradiation and the radioprotective effect of the membrane stabilization agent lidocaine [11]. As a consequence of the intracellular signaling and secretion, our scintigraphic results verified that radiation induced damage of the acini's water secretion ability and that lidocaine prevented this impairment. This conclusion was also supported by our ultrastructural findings of damage to the basolateral cell membrane, a key site for radiation-induced damage of the Na^+^/K^+^/2Cl^–^ cotransporter. Lidocaine's cell membrane stabilization may therefore explain its radioprotective effect on saliva secretion.

Interestingly, saliva total protein concentration was not affected by radiation in our study. The result is in accordance with previous studies and leakage of serum proteins into saliva is one possible explanation. [23], [24]Whereas most water secretion occurs subsequent to muscarinic receptor activation, protein secretion follows β-adrenergic receptor activation. β-adrenergic receptor activation induces a Gs-protein-mediated cyclic-AMP signal, which evokes protein secretion in a dose-dependent manner [21]. According to a four-phase pattern of radiation-induced damage, in phase one (within 10 days after irradiation), alteration of the plasma membrane interferes with activation of the muscarinic receptor, with Gq-protein, or with both but does not impair interactions with the β-adrenergic receptor and the Gs-protein [4], [8], [9]. Our findings not only are consistent with this hypothesis but also confirm the crucial role of this pathway as a potential target of radioprotection. Our data also verified the fact that in the early stage after irradiation, water secretion is specifically and severely damaged whereas protein alteration remains negligible [4], [9].

Based on the serum level curve of lidocaine reported in our previous study[1], we used systemic delivery of lidocaine in the present study because it was feasible to choose the optimal dose and application time point. However one question raised by our results is whether the systemic application of lidocaine will also protect tumors from radiation. Although we have not investigated this issue yet, previous studies showed that the membrane-binding agents provide cell protection under the normoxic conditions that dominate in normal salivary gland tissue but sensitize cells to radiation under the hypoxic conditions found in solid head and neck tumors [25]. Furthermore, the selective radioprotection of muscarinic agonist-induced water secretion elucidated in the present study also suggests that this drug is safe. Anyway, in the future studies, local delivery of lidocaine through salivary ducts would be more desirable for the possible translation to clinical application.

There are several limitations in the present study to be addressed. First, we only investigated the radioprotective effect of lidocaine in early-stage damage. Based on our previous studies, we chose one week after radiation as the study time-point, which can provide comparable histopathological changes that indicate the overall course of functional changes of salivary glands through one month after radiation[11], [26]. However the late-stage effects are attributed by lack of repopulation from radiation induced ductal progenitor cells damage[2]. Whether the radioprotection of muscarinic agonist-induced water secretion by lidocaine plays a role in the long-term aspect still needs to be revealed by an alternative research design in future studies. Second, although our study revealed the protective effect of lidocaine on the muscarinic agonist-induced water secretion in irradiated salivary glands, there are still a couple of issues to be addressed to further explore the exact mechanisms, including the involvement of the muscarinic receptor axis in the radioprotective profile, and the effect of lidocaine on microvasculature and innervations of the glands. These future investigations will lead us to better understand the pharmacological mechanism of lidocaine at cellular and molecular level.

## Conclusions

In summary, the in vivo and in vitro studies presented here demonstrate that impairment of the intracellular signal transduction induced by binding of muscarinic agonists and membrane receptors is an essential mechanism of early-stage radiogenic salivary gland dysfunction. Furthermore, for the first time, we have provided evidence of the radioprotective effect of lidocaine on salivary gland cells' capacity for muscarinic agonist-induced water secretion, which may explain the protection profile of lidocaine on salivary glands during irradiation.
